# Low-Dose Dobutamine Stress Echocardiography for the Early Detection of Pulmonary Arterial Hypertension in Selected Patients with Systemic Sclerosis Whose Resting Echocardiography Is Non-Diagnostic for Pulmonary Hypertension

**DOI:** 10.3390/jcm10173972

**Published:** 2021-09-02

**Authors:** Loukianos S. Rallidis, Konstantina Papangelopoulou, Georgios Makavos, Christos Varounis, Anastasia Anthi, Stylianos E. Orfanos

**Affiliations:** 1 Second Department of Cardiology and Pulmonary Hypertension Clinic, Attikon Hospital, School of Medicine, National & Kapodistrian University of Athens, 16462 Athens, Greece; kpapanel@gmail.com (K.P.); gmakavos@hotmail.com (G.M.); varounis@hotmail.com (C.V.); 2 Second Department of Critical Care and Pulmonary Hypertension Clinic, Attikon Hospital, School of Medicine, National & Kapodistrian University of Athens, 16462 Athens, Greece; anastasia.anthi1@gmail.com; 3 First Department of Critical Care and Pulmonary Hypertension Center, Evangelismos General Hospital, Medical School, National & Kapodistrian University of Athens, 10676 Athens, Greece; stylianosorfanosuoa@gmail.com

**Keywords:** dobutamine stress echocardiography, pulmonary arterial hypertension, systemic sclerosis

## Abstract

Background: Dobutamine stress echocardiography (DSE) has limited application in systemic sclerosis (SSc). We examined DSE usefulness in revealing pulmonary arterial hypertension (PAH) in selected SSc patients whose resting echocardiography for pulmonary hypertension (PH) was non-diagnostic. Methods: Forty SSc patients underwent right heart catheterization (RHC) and, simultaneously, low-dose DSE (incremental doses up to 20 μg/kg/min). Inclusion criteria were: preserved left and right ventricular (RV) function (tricuspid annulus plane systolic excursion [TAPSE] ≥ 16 mm and tissue Doppler imaging-derived systolic velocity of tricuspid annulus [RVS’] > 10 cm/s), normal pulmonary function tests, and baseline maximal tricuspid regurgitation (TR) velocity of 2.7–3.2 m/s. Results: Of 36 patients who completed DSE, resting RHC diagnosed PAH in 12 patients (33.3%). At 20 μg/kg/min, patients with PAH had higher TR velocity, higher pulmonary arterial pressure measured by RHC, and lower RV inotropic response compared with patients without PAH. A cut-off value of maximal TR velocity >3.1 m/s had a sensitivity of 80%, a specificity of 84.2%, and an accuracy of 82.4% for the detection of PAH. Conclusions: Low-dose DSE has a satisfactory diagnostic accuracy for the early detection of PAH in highly selected SSc patients whose baseline echocardiographic measurements for PH lie in the gray zone.

## 1. Introduction

Pulmonary arterial hypertension (PAH) is a common complication of systemic sclerosis (SSc) [1,2] associated with increased morbidity and mortality [3]. The early diagnosis of PAH in SSc is crucial for the initiation of treatment in order to delay clinical deterioration and improve survival [4,5,6,7]. Although a diagnosis of pulmonary hypertension (PH) is established by right heart catheterization (RHC), the measurement of maximal tricuspid regurgitation (TR) velocity at rest by Doppler echocardiography is an important tool for the non-invasive estimation of pulmonary arterial pressure (PAP) [2]. However, it has been demonstrated that in SSc patients, detection of PH by resting echocardiography is often inaccurate [8].

Exercise stress echocardiography has been applied in the past for the identification of PH in SSc patients [9], but the lack of standardization and of prospective confirmatory data has downgraded its role for the detection of PH in the 2015 ESC/ERS guidelines [2]. There are very few data on the role of dobutamine stress echocardiography (DSE) in SSc or PAH patients either for the detection of wall motion abnormalities of the left ventricle (LV) or for the assessment of right ventricular (RV) inotropic reserve [10,11,12].

Recently, a lower cut-off value, i.e., mean PAP > 20 mmHg measured by RHC along with pulmonary vascular resistance (PVR) ≥ 3 Wood Units and pulmonary arterial wedge pressure (PAWP) ≤ 15 mmHg, for the definition of PAH was proposed [13] and has already been adopted by the ESC guidelines for Grown-Up Congenital Heart Disease [14]. In the present study, we explored the potential value of low-dose DSE to identify PAH in selected asymptomatic or mildly symptomatic patients with SSc whose resting echocardiography was equivocal for the presence of PH, i.e., the systolic PAP (sPAP) was between 35 and 45 mmHg, which corresponds to a maximal TR velocity between 2.7 and 3.2 m/s.

## 2. Material and Methods

### 2.1. Study Population

We prospectively studied 40 patients with SSc referred to the PH Clinic of Attikon University Hospital for PAH screening. All patients underwent a detailed clinical and laboratory work-up, as described below.

Inclusion criteria were the following: (1) baseline maximal TR velocity ranging between 2.7 and 3.2 m/s, (2) preserved LV [ejection fraction (EF) > 55%] and RV function (tricuspid annulus plane systolic excursion [TAPSE] ≥ 16 mm and tissue Doppler imaging (TDI)-derived systolic velocity of tricuspid annulus [RVS’] > 10 cm/s), (3) no previous ischemic or valvular heart disease or echocardiographic findings of diastolic dysfunction suggestive of elevated LV filling pressures, (4) sinus rhythm, and (5) forced expiratory volume in 1 s (FEV_1_) ≥ 55% of predicted normal and total lung capacity (TLC) ≥ 60% of predicted normal [15].

The study was approved by the Ethics ommittee of our hospital, and all subjects gave signed informed consent.

### 2.2. Resting Echocardiography

A resting echocardiography study was performed during the subjects’ first visit to the PH clinic with a Vivid 9 ultrasound system (GE Medical Systems, Horten, Norway) in line with the latest guidelines of the American Society of Echocardiography and the European Association of Cardiovascular Imaging [16]. Assessment of diastolic function was made by measuring the peak E and A transmitral velocities with pulse wave Doppler and the e’ wave at the lateral mitral annulus aspect and septal basal regions with TDI [17]. TAPSE, RVS’, and maximal TR velocity were also recorded. Right atrial pressure (RAP) was calculated from the inferior vena cava diameter and its respiratory variation [18]. sPAP was estimated by using the modified Bernoulli equation: sPAP = 4 × (maximal TR velocity)^2^ + RAP.

### 2.3. Right Heart Catheterization

All patients underwent RHC the day of DSE. RHC was performed 20–30 min prior to DSE in the catheterization laboratory by a single operator (AA) using an echo-guided right internal jugular vein access. Local anesthesia was performed by s.c. lidocaine, and a triple lumen Edwards Swan–Ganz catheter of 7 F was inserted. The following measurements were performed: RAP, PAP (systolic, diastolic; a mean was then calculated), PAWP, and cardiac output (CO) by thermodilution. PVR and cardiac index (CI) were subsequently calculated. For diagnosing PAH, we applied the 2019 proposed criteria [13], i.e., mean PAP > 20 mmHg along with PVR ≥ 3 Wood Units and PAWP ≤ 15 mmHg.

### 2.4. Low-Dobutamine Stress Echocardiography

Before the initiation of dobutamine infusion, a resting echocardiographic study was performed in the catheterization laboratory with the patient lying supine. Afterwards, the infusion of dobutamine was started with parallel echocardiographic imaging and continuous hemodynamic recording. Echocardiographic imaging was focused on the 4-chamber view to visualize the tricuspid and mitral valves. Dobutamine was given as a continuous infusion at incremental doses of 5, 10, 15, and a maximum of 20 μg/kg/min. Each infusion stage lasted for 3 min, and during the last 30 s of each stage, both echocardiographic and hemodynamic measurements were performed. Echocardiographic assessment included measurements of maximal TR velocity, transmitral E and A velocities, RVS’, and TAPSE. Hemodynamic assessment comprised measurements of systolic, diastolic, and mean PAP as well of PAWP and CO. All echocardiographic measurements were obtained by a single operator (LSR) blinded to the RHC results. For comparisons of baseline echocardiographic values with RHC measurements, echocardiographic values obtained by resting echocardiography prior to the RHC were used.

### 2.5. Six-Min Walk Test

A 6-min walk test (6MWT) was conducted on a straight pre-marked corridor under the supervision of an expert nurse. Total walking distance and percutaneous oxygen saturation were measured at the end of the test. The appearance of relevant symptoms (e.g., fatigue, dyspnea) was also noted.

### 2.6. Pulmonary Function Tests and Lung Imaging

All patients had chest X-ray, high-resolution computed tomography (HRCT) of lungs, and pulmonary function tests (PFTs), i.e., FEV_1_ and TLC.

### 2.7. Biochemical Measurements

Fasting blood was drawn the day of catheterization for N-terminal pro-B-type natriuretic peptide (NT-proBNP), high-sensitivity C-reactive protein (hsCRP), and routine hematological and biochemical measurements.

### 2.8. Statistical Analysis

Continuous variables are displayed as means ± standard deviation (SD), while non-normally distributed variables are presented as medians and interquartile ranges. The Student’s t test was applied for independent samples to compare means for normally distributed variables, and the Mann–Whitney test was used for non-normally distributed variables. Categorical data are presented as proportions, and associations between them were tested by the chi-square test.

Receiver Operating Characteristic (ROC) analysis was used to identify the optimal cut-off value (best combination of sensitivity and specificity) that distinguished patients with PAH. Sensitivity, specificity, disease prevalence, positive and negative predictive value, and accuracy were calculated and expressed as percentages. Confidence intervals (CIs) for sensitivity, specificity, and accuracy were calculated as “exact” Clopper–Pearson CIs, and the CIs for the likelihood ratios were calculated with the “Log method” [19]. CIs for the predictive values were estimated as the standard logit CIs [20].

A *p* value < 0.05 was used to indicate statistical significance. The Statistical Package for the Social Sciences (SPSS) release 26.0 (SPSS Inc., Chicago, IL, USA) was used.

## 3. Results

During the period 2018–2020, 40 of 150 patients with SSc referred to the PH clinic fulfilled the inclusion criteria and agreed to participate. Of those, two had a poor-quality TR Doppler signal during dobutamine infusion, and two developed a hypotensive response (symptomatic drop of systolic blood pressure ≥ 20 mmHg from baseline or previous level of infusion) leading to premature termination of DSE [21]. This left us with 36 patients, of whom 29 (80%) had diffused cutaneous SSc, and 7 (20%) limited cutaneous SSc (CREST syndrome). Table 1 presents the characteristics of the patients. All patients were women. The mean duration of disease from first symptoms related to SSc until the first visit was 7.5 ± 4.2 years.

**Figure 1:**
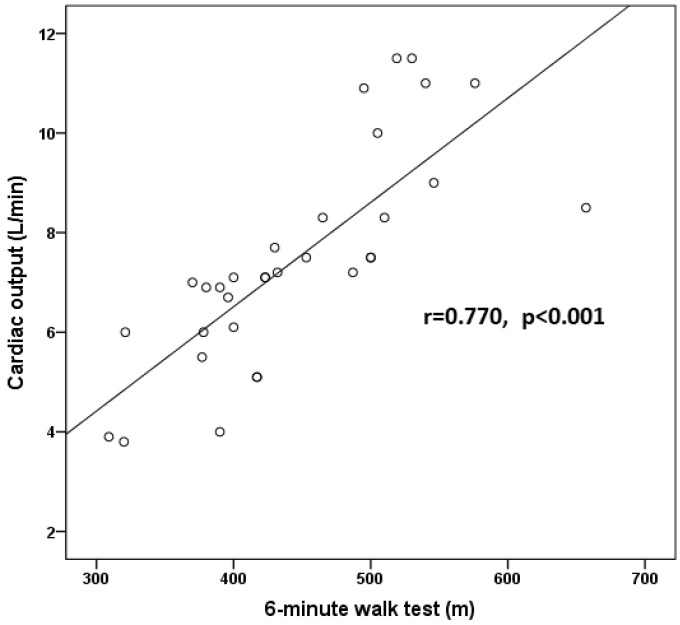
Scatter plot correlation of cardiac output measured with right heart catheterization at 20 μg/kg/min of dobutamine infusion with 6-minute walk test.

Table 2 shows patients’ features according to the presence of PAH. Patients with PAH at the dose of 20 μg/kg/min had higher maximal TR velocity, higher sPAP (measured by both echocardiography and RHC), and lower RV inotropic response [indicated by lower Δ (RVS’) and Δ (TAPSE)] and systolic blood pressure compared to patients without PAH. CO at the dose of 20 μg/kg/min was also lower in patients with PAH, but the difference did not reach statistical significance. There was no difference between the two groups in NT-proBNP and hsCRP levels as well in the E/e’ ratio. CO was positively correlated with 6MWT (r = 0.777, *p* < 0.001) (Figure 1).

A ROC curve analysis of maximal TR velocity discriminated the patients as those with RHC-validated PAH and those without it (AUC = 0.852 with 95%CI: 0.705–1.000, *p* = 0.001). A cut-off value of maximal TR velocity at 20 μg/kg/min > 3.1 m/s had a sensitivity of 80%, a specificity of 84.2%, and an accuracy of 82.4% for the detection of PAH established by RHC (Table 3).

## 4. Discussion

Ιn the present study, we demonstrated that DSE has a satisfactory diagnostic accuracy for the early detection of PAH in selected patients with SSc, i.e., asymptomatic or mildly symptomatic patients with equivocal resting echocardiographic study for PH and low probability of having PH due to left heart or lung disease. In particular, we found that maximal TR velocity during low-dose DSE > 3.1 m/s had a diagnostic accuracy of 82.4%, with a specificity of ~84% and a moderate sensitivity of 80% for the identification of PAH confirmed by RHC.

To our knowledge, this is the first study exploring the usefulness of DSE in SSc for the detection of PAH. Another novel element of our study is the use of the recently proposed cut-off value of mean PAP > 20 mmHg for the diagnosis of PAH [13].

There are few data on the role of DSE in PAH or SSc patients, mainly focused on the assessment of RV inotropic reserve. Ghio et al. [11] showed in 55 patients with PAH that during low-dose DSE, the increase in CO was related to an improvement in longitudinal RV function and that the increase in pulmonary pressure was mainly a consequence of the chronotropic response to dobutamine. Sharma et al. [12] demonstrated that during low-dose DSE, RV contractile reserve assessed by the change in TAPSE and S’ waves (i.e., peak stress minus rest value) was markedly depressed in PAH patients compared to controls and that reduced RV contractile reserve was associated with reduced exercise capacity in PAH patients. We also found that patients with PAH had a lower inotropic RV response with DSE compared to patients without PAH, but this was not associated with a decreased exercise capacity, since 6MWT was similar in these two subgroups. This may be due to the low sensitivity of 6MWT to reveal functional impairment in asymptomatic or mildly symptomatic PAH patients [22].

There are several previous attempts using various strategies or algorithms for early PAH diagnosis in SSc [5,8,23]. Investigators from the DETECT trial (8) developed an algorithm including six variables from clinical evaluation and non-invasive tests plus two echocardiographic variables (right atrium area and maximal TR velocity). This DETECT-based algorithm demonstrated a high overall sensitivity (96%) but poor specificity (48%) in the detection of PAH validated with RHC in SSc patients at high risk for PAH. In another study, Mukerjee et al. [23], in a population of 137 SSc patients of whom 52 had pulmonary fibrosis, applied various TR velocity thresholds and tested their sensitivity and specificity to detect PAH. The best specificity (97%) was found at a cut-off of maximal TR velocity >3.2 m/s but at the expense of sensitivity (47%) for the diagnosis of PAH. The diagnostic performance of DSE in our study cannot be compared with that of these strategies which were tested in SSc patients at high risk for PAH as opposed to the SSc population of our study with low (predominantly) or low to intermediate risk. In addition, for the definition of PAH, we used a lower cut-off value of mean PAP, i.e., >20 mmHg.

The idea to assess pulmonary circulation under dynamic stress has been around for many years. In particular, exercise echocardiography has been used for more than 15 years in SSc [9,24,25,26,27,28], but the lack of standardization has limited its application for the diagnosis of PH [2,9]. So far, DSE has not been applied for the early detection of PH in SSc patients.

Dobutamine compared to exercise echocardiography has the advantage that it can be administered to patients unable to perform exercise due to orthopedic problems and it is technically less demanding. Dobutamine as a stressor increases CO by its inochronotropic action, and it has been suggested that it induces pulmonary vasodilatation. Lau et al. [29] applied low-dose DSE (up to 20 μg/kg/min) in 16 PAH patients and assessed non-invasively the pressure–flow relationships of the pulmonary circulation. They found that subjects with PAH had markedly elevated mean PAP-CO slopes (5.1 ± 2.5 mmHg/L/min) compared to healthy subjects (1.1 ± 0.7 mmHg/L/min), and this was associated with poor New Work Heart Association functional status. The authors proposed that an abnormal mean PAP-CO response may be an early sign of pulmonary vascular disease in high-risk for PAH subjects. In accordance with these findings, Kafi et al., in an invasive study of 11 patients with idiopathic PAH, reported a pooled dobutamine-induced mean PAP/CO slope of 8.3 mmHg/L/min) [30].

Our study was based on the hypothesis that in the early stages of PAH, resting pulmonary pressure measurements may be normal or near normal due to the pulmonary vasculature reserve. However, dobutamine-induced increase in pulmonary blood flow may overcome the vasculature reserve, reveal a latent circulatory dysfunction, increase PAP, and unmask PH in SSc patients.

The development of PH in SSc patients is multifactorial. Several factors may be implicated in resting or dobutamine-induced PH in SSc. Besides pulmonary vascular remodeling, diastolic dysfunction and interstitial lung fibrosis may be involved [31]. In our study, we excluded SSc patients with interstitial lung fibrosis based on HRCTs and PFTs or diastolic dysfunction associated with elevated LV filling pressures. Thus, DSE-induced elevation in sPAP was mainly attributed to impaired pulmonary vascular reserve as an early expression of PAH.

## 5. Study Limitations

The main limitation of our study is the relatively small sample size. Therefore, larger studies are needed to replicate our results and define the exact role of DSE in SSc patients. Another limitation is that DSE was used in a highly selected subgroup of SSc patients, and therefore its value for revealing PAH cannot be generalized for all SSc patients with equivocal resting echocardiography for PH.

## 6. Conclusions

Low-dose DSE has a satisfactory diagnostic accuracy in revealing PAH in selected asymptomatic or oligosymptomatic patients with SSc whose resting echocardiography for PH is non-diagnostic. Therefore, it may be a useful “noninvasive” technique in SSc patients with low or low to intermediate probability of PAH and equivocal echocardiography for PH, particularly in hospitals without facilities for RHC. If DSE is suggestive of PAH, then RHC should be performed. However, it should be emphasized that DSE has no place as a diagnostic tool in SSc patients with intermediate-to-high or high probability of PAH who require referral to a PH clinic for RHC and initiation of appropriate treatment if PAH is confirmed.

## Figures and Tables

**Table 1:** Characteristics of the examined patients with systemic sclerosis (*n* = 36).

Characteristics	
Age (years)	60.5 ± 11.0
Females, n (%)	36 (100)
Body mass index (kg/m^2^)	25.4 ± 3.2
Duration of disease (years)	7.5 ± 4.2
FEV_1_, % predicted	75.5 ± 9.8
TLC, % predicted	75.3 ± 9.1
6-min walk test (m)	444.0 ± 73.9
**Baseline echocardiography at the day of right heart catheterization**	
Ejection fraction of left ventricle (%)	63.9 ± 3.6
Left atrial volume index (mL/m^2^)	27.0 ± 4.3
E/e’ (average)	9.8 ± 2.3
TR velocity (m/s)	2.78 ± 0.11
sPAP (mmHg)	34.6 ± 3.5
TAPSE (mm)	21.6 ± 4.0
RVS’ (cm/s)	12.5 ± 2.8
Right atrial pressure (mmHg)	3.3 ± 1.0
**Right heart catheterization (resting)**	
Right atrial pressure (mmHg)	3.9 ± 1.9
PAWP (mmHg)	7.2 ± 2.5
Mean PAP (mmHg)	19.0 ± 5.8
sPAP (mmHg)	29.8 ± 6.8
Cardiac output (L/min)	5.0 ± 1.3
Cardiac index (L/min/m^2^)	2.9 ± 0.67
PVR (Wood Units)	2.46 ± 1.5
**Biochemistry**	
hsCRP (mg/L)	3.25 (3.1–6.6)
NT-proBNP (pg/mL)	198.7 ± 177.7
Creatinine (mg/dL)	0.82 ± 0.28
Hematocrit (%)	39.1 ± 2.6

FEV1 = forced expiratory volume in 1 s, TLC = total lung capacity, PVR = pulmonary vascular resistance, sPAP = systolic pulmonary arterial pressure, PAWP = pulmonary arterial wedge pressure, TAPSE = tricuspid annular plane systolic excursion, TR = tricuspid regurgitation, NT-proBNP = N-terminal pro-B-type natriuretic peptide, RV = right ventricle, hsCRP = high-sensitivity C-reactive protein, RVS’ = tissue Doppler imaging-derived systolic velocity of tricuspid annulus.

**Table 2:** Characteristics of the patients with systemic sclerosis according to the presence of pulmonary arterial hypertension (PAH) [mean PAP > 20 mmHg].

Characteristics	PAH (*n* = 12)	No PAH (*n* = 24)	*p* Value
Age (years)	57.8 ± 9.5	61.9 ± 111.0	0.233
Body mass index (kg/m^2^)	24.3 ± 4.0	26.1 ± 2.8	0.154
Duration of disease (years)	6.7 ± 2.9	7.7 ± 4.6	0.589
FEV_1_, % predicted	74.5 ± 10.8	76.2 ± 9.9	0.596
TLC, % predicted	75.7 ± 7.8	74.7 ± 7.1	0.756
6-min walk test (m)	454.2 ± 82.9	452.5 ± 77.1	0.958
**Baseline echocardiography at the day of right heart catheterization**			
Ejection fraction of left ventricle (%)	62.4 ± 4.4	64.4 ± 3.2	0.130
Left atrial volume index (mL/m^2^)	25.7 ± 5.1	27.6 ± 3.8	0.231
E/e’ (average)	9.7 ± 2.0	9.8 ± 2.5	0.828
TR velocity (m/s)	2.84 ± 0.11	2.75 ± 0.07	0.005
sPAP (mmHg)	37.1 ± 4.1	33.4 ± 2.3	0.005
TAPSE (mm)	19.4 ± 4.2	22.7 ± 3.6	0.022
RVS’ (cm/s)	11.1 ± 2.9	13.2 ± 2.5	0.030
Right atrial pressure (mmHg)	4.5 ± 2.2	3.4 ± 1.4	0.121
**Right heart catheterization (resting)**			
Right atrial pressure (mmHg)	4.4 ± 2.3	3.7 ± 1.9	0.377
PAWP (mmHg)	7.0 ± 2.4	7.5 ± 2.5	0.619
Mean PAP (mmHg)	25.1 ± 2.9	15.4 ± 2.9	<0.001
sPAP (mmHg)	37.5 ± 3.5	25.6 ± 3.9	<0.001
Cardiac output (L/min)	4.4 ± 0.9	5.5 ± 1.4	0.081
Cardiac index (L/min/m^2^)	2.6 ± 0.5	3.1 ± 0.7	0.050
PVR (Wood Units)	4.2 ± 1.1	1.5 ± 0.5	<0.001
**Dobutamine infusion (values at 20 μg/kg/min)**			
Echo measurements			
E/e (average)	7.0 ± 1.5	10.1 ± 3.4	0.077
TR velocity (m/s)	3.1 ± 0.27	2.7 *±* 0.32	<0.001
sPAP (mmHg)	41.9 ± 6.3	32.9 ± 7.3	<0.001
TAPSE (mm)	20.7 ± 2.8	26.4 ± 4.2	<0.001
RVS’ (cm/sec)	17.6 ± 5.1	21.5 ± 5.1	0.038
Δ (TAPSE) [mm]	1.4 ± 2.5	3.6 ± 3.1	0.034
Δ (RVS’) [cm/sec]	4.9 ± 4.1	8.2 ± 4.2	0.030
Hemodynamic measurements			
Right atrial pressure (mmHg)	4.3 ± 2.3	3.7 ± 1.8	0.377
PAWP (mmHg)	7.0 ± 1.8	5.7 ± 3.2	0.249
Mean PAP (mmHg)	26.9 ± 3.9	15.1 ± 3.8	<0.001
sPAP (mmHg)	43.9 ± 7.7	27.9 ± 7.9	<0.001
Cardiac output (L/min)	6.6 ± 1.9	8.1 ± 2.2	0.081
Cardiac index (L/min/m^2^)	3.9 ± 1.2	4.5 ± 1.3	0.250
PVR (Wood Units)	4.2 ± 1.1	1.6 ± 0.5	<0.001
sBP (mmHg)	126.1 ± 16.0	143.8 ± 22.1	0.028
Heart rate (beats/min)	116.4 ± 15.6	111.1 ± 21.6	0.457
**Biochemistry**			
hsCRP (mg/L)	3.29 (3.1–11.2)	3.19 (3.1–5.1)	0.754
NT-proBNP (pg/mL)	192.5 ± 201.4	209.6 ± 176.9	0.801
Creatinine (mg/dL)	0.78 ± 0.17	0.86 ± 0.30	0.333
Hematocrit (%)	39.7 ± 1.3	38.5 ± 3.1	0.241

sBP = systolic blood pressure, other abbreviations as in Table 1.

**Table 3:** Diagnostic test evaluation of maximal tricuspid regurgitant velocity >3.1 m/s at 20 μg/kg/min of dobutamine infusion during stress echocardiography.

	Point Estimate	95%CI
Sensitivity (%)	80.0	44.4–97.5
Specificity (%)	84.2	60.4–96.6
LR+	5.07	1.71–14.97
LR−	0.24	0.07–0.83
PPV (%)	79.2	56.4–91.9
NPV (%)	84.8	61.4–95.1
Accuracy (%)	82.4	63.8–93.9

CI = confidence interval, LR+ = positive likelihood ratio, LR− = negative likelihood ratio, PPV = positive predictive value, NPV = negative predictive value.

## Data Availability

The data presented in this study are available on reasonable request from the corresponding author (L.S.R.). The data are not publicly available due to privacy and ethical issues.
